# 267. Delayed Pulmonary Tuberculosis (PTB) Diagnosis During the COVID-19 Pandemic: A Case Series

**DOI:** 10.1093/ofid/ofac492.345

**Published:** 2022-12-15

**Authors:** Mikhail Khaitov, Nadim Salomon, David Perlman, Sarah Humphreys

**Affiliations:** Mount Sinai Hospital, Flushing, New York; Mount Sinai Beth Israel, New York, New York; Mount Sinai Beth Israel, New York, New York; Mount Sinai Hospital, Flushing, New York

## Abstract

**Background:**

The COVID-19 pandemic has been associated with underreporting of pulmonary tuberculosis (PTB). Overlapping risk factors, clinical features, and chronicity of post COVID-19 sequelae can lead to attribution of respiratory disease solely to COVID-19 and result in delayed recognition of PTB.

**Methods:**

Identification of inpatients with both sputum (spu) culture (clt) positive TB and SARS-CoV-2 + PCR was achieved using data extraction tool Slicer Dicer Epic system during 3/2020-1/2022 followed by a retrospective review of electronic medical records. We defined COVID-PTB as a patient (pt) who had positive spu clt for TB ≤ 12 months (mos) following a COVID-19 diagnosis.

**Results:**

8 pts had both PTB and COVID-19. Two had PTB > 5 mos prior to, and one had PTB > 12 mos after COVID-19. 3/8 were promptly suspected to have PTB [1 with right upper lobe (RUL) infiltrate; 1 RUL cavitary infiltrate; 1 with miliary nodules and cavities]; in these 3, spu was tested for MTB ≤ 48 hours (h) (hrs) of presentation. 5/8 had COVID-PTB. All 5 had fever and/or respiratory symptoms and ≥ 1 risk factors for TB identified at the time of presentation with COVID-19. Spu was tested for MTB in 48 hs in 1 with RUL cavitary infiltrate, and 5 days after chest CT findings of apical densities and scattered nodules in another. In the remaining 3, spu testing was delayed a median of 36 days (range, 8-133) after initial TB consistent CT findings (LUL opacity and nodular densities in 1; RLL cavitary infiltrate in 1; and clustered RML nodules in 1) and despite immigration from high burden TB countries in all 3; known LTBI in 2; diabetes in 1, and immunosuppressive therapies in 2.

**Conclusion:**

We observed a delay in sputum collection after COVID-19 diagnosis in the presence of epidemiological risk factors for TB disease and clinical features consistent with PTB. Lack of familiarity with the range radiological TB features, a diagnostic bias towards more typical radiographic (e.g., cavities, miliary patterns) and COVID-19 anchoring bias may have contributed to delayed PTB diagnosis. PTB should be considered, when clinically appropriate, in the setting of COVID-19 or apparent post COVID-19 sequelae.

**Disclosures:**

**All Authors**: No reported disclosures.

